# PTCL, NOS: An update on classification, risk-stratification, and treatment

**DOI:** 10.3389/fonc.2023.1101441

**Published:** 2023-02-09

**Authors:** Jonathan Weiss, John Reneau, Ryan A. Wilcox

**Affiliations:** ^1^ Department of Internal Medicine, Division of Hematology and Oncology, University of Michigan, Ann Arbor, MI, United States; ^2^ Department of Medicine, Division of Hematology, The Ohio State University Comprehensive Cancer Center, Columbus, OH, United States

**Keywords:** PTCL-NOS, PTCL, GATA-3, risk stratification, classification, treatment

## Abstract

The peripheral T-cell lymphomas (PTCL) are relatively rare, heterogeneous, and therapeutically challenging. While significant therapeutic gains and improved understanding of disease pathogenesis have been realized for selected PTCL subtypes, the most common PTCL in North America remains “not otherwise specified (NOS)” and is an unmet need. However, improved understanding of the genetic landscape and ontogeny for the PTCL subtypes currently classified as PTCL, NOS have been realized, and have significant therapeutic implications, which will be reviewed here.

## Introduction

Historically, treatment paradigms utilized successfully in the management of many aggressive B-cell lymphomas – namely, multiagent, anthracycline-based regimens, including CHOP – have been empirically applied, perhaps unjustifiably (1), to the T-cell lymphomas. With one notable exception (2), attempts to improve outcomes by “adding to” or “replacing” anthracycline-based regimens have failed, and most T-cell lymphoma patients will succumb to their lymphoma, or complications related to its treatment, within a few years of diagnosis. Indeed, most of the T-cell lymphomas have been, and remain, a challenging and unmet medical need (3).

The sheer geographic, clinical, histopathologic, molecular, and genetic heterogeneity of the more than 25 peripheral T-cell lymphoma (PTCL) subtypes recognized by the WHO have frustrated efforts to improve outcomes for patients afflicted with these mature T-cell derived non-Hodgkin lymphomas (NHL) (4). Not surprisingly then, PTCL diagnosis and classification are challenging, as demonstrated by the relatively high-rate (≈33%) of reclassification following “expert” hematopathology review (5), after which ≈25-40% will remain “unspecified” (6, 7). At the dawn of the 21^st^ century, the PTCL were viewed as the “next, and largely unexplored, frontier in lymphoma management” (8). Twenty years later, significant gains, driven by collaborative and multidisciplinary efforts, have improved our understanding of the PTCL generally, and PTCL, not otherwise specified (PTCL, NOS) specifically. The new frontier in lymphoma management spawned by these efforts is dominated by novel therapeutic approaches, as evidenced by the more than 100 ongoing clinical trials investigating novel agents. Despite these significant advances, and evidence to support a cautiously optimistic outlook (9), the PTCL, NOS remain a diagnostic and therapeutic challenge, and are the subject of this review.

## Epidemiology

In North America and Europe, with the exception of American Indians, PTCL, NOS is the most common PTCL subtype, accounting for ≈30% of PTCL, and is twice as prevalent as either angioimmunoblastic T-cell lymphomas (AITL) or anaplastic large cell lymphomas (ALCL) (6, 7, 10). The median age at diagnosis is 60 years, and males are more commonly affected, with a male-to-female ratio of 1.9:1 (6). A history of celiac disease, psoriasis, and a family history of any hematologic malignancy in a first-degree relative are risk factors for PTCL, NOS, with adjusted odds ratios ranging from ≈2-9, whereas a history of allergies and moderate alcohol consumption are associated with a decreased risk (11). While long-term (>40 years) cigarette smoking is a significant risk factor in PTCL overall, this association did not reach statistical significance for PTCL, NOS (11). Immune suppression may also confer an increased risk of PTCL, NOS, possibly including those derived from cytotoxic T cells (12, 13), and a history of prior immunosuppressive therapies has been reported in 3.8% of PTCL, NOS patients (14). Consistent with the importance of immune surveillance, PTCL, NOS is observed following solid organ transplant, and accounts for approximately one-third of T-cell derived post-transplant lymphoproliferative disorders (15). Deleterious mutations or copy number alterations/structural variants are recurrently observed in genes required for immune surveillance (or evasion), including MHC class I, β2M, and PD-L1 (16, 17). When observed, PTLD (PTCL, NOS) often occur “late” (>5 years post-transplant), frequently involve extranodal sites, and are often, but not always, EBV-associated, regardless of the time of onset following transplant (15), and are seemingly associated with a genetic landscape reminiscent of that observed in PTCL, NOS generally (18). Not surprisingly, T-cell derivation is an adverse prognostic factor on multivariate analysis in the setting of PTLD (19). With the possible exception of PTLD, EBV more commonly affects B-cells within the tumor microenvironment, and rarely infects malignant T cells, at least in the West (14). However, EBV associated PTCL, NOS is more common in Asia, where EBV infection is associated with inferior outcomes (20). While previously classified as a subtype of PTCL, NOS, in the current WHO classification these lymphomas are now classified as nodal EBV-positive T and NK-cell lymphomas (4).

## Natural history and risk-stratification

The majority of patients diagnosed with PTCL, NOS present with advanced-stage (III/IV) disease, usually with nodal or nodal/extranodal involvement (14). Involvement of multiple extranodal sites or exclusively extranodal involvement are less common, being observed in 29% and 13% of patients, respectively (14). Common extranodal sites of involvement, observed in at least 10% of patients, include the spleen, bone marrow, liver, and skin (14). Associated complications, including hypercalcemia, hypogammaglobulinemia, and hemophagocytic lymphohistiocytosis (HLH) are observed in fewer than 10% of patients (14).

Approximately 30% of patients remain alive and disease free 24 months following initial diagnosis and treatment (14, 21), and achieving this landmark is a surrogate for overall survival (22, 23). Among PTCL, NOS patients who experienced disease progression or recurrence within 24 months of diagnosis, the 3-year overall survival from the time of progression was 19.4%. In stark contrast, event-free survival at 24 months (EFS24) was associated with a 3-year overall survival, from the time EFS24 was achieved, of 84.6% (22). As with most aggressive NHL, advanced age and poor performance status are adverse prognostic factors (6, 24–26), as are an elevated LDH (14, 24, 25), bone marrow involvement (14, 24), bulky (≥10 cm) disease (14), thrombocytopenia (14), lymphopenia (27), neutrophilia (26), hypoalbuminemia (26), and a high proliferative index (Ki67 ≥80%) (25). These prognostic variables have been variously combined to form prognostic indices, stratifying patients into low- and high-risk groups, with EFS24 ranging from ≈20% to ≈50%, respectively. While still utilized, one suspects that these indices may be supplanted by, or at least incorporate, ontologically and/or genetically based approaches for risk-stratification in the near future.

## Disease ontology and classification

PTCL, NOS are morphologically heterogeneous, ranging from sheets of intermediate- to large-sized cells that are relatively devoid of an inflammatory environment (i.e. immunologically “cold”) to those that are polymorphous and enriched for a range of inflammatory cells (i.e. immunologically “hot”) (28, 29), including epithelioid histiocytes [in “Lennert’s lymphomas” (30)]. T-cell specific antigens (e.g. CD2, CD3, CD4) are commonly expressed, while CD4 and/or CD8 expression is variable, and not always associated with the presumed cell of origin (31). The majority (>90%) of PTCL, NOS are derived from mature αβ T-cells. T-cell lymphomas derived from γδ T cells classically involve specific extranodal sites, but when classified as PTCL, NOS, transcriptionally resemble extranodal NK/T cell lymphomas (32).

Early transcriptional profiling efforts highlighted PTCL, NOS heterogeneity, with subsets transcriptionally resembling either ALK- ALCL or AITL, while others were transcriptionally disparate (33, 34). Subsequent transcriptional profiling efforts demonstrated that ≈15% of PTCL, NOS cases transcriptionally resemble follicular helper T-cell (T_FH_)-derived PTCL (35). Consequently, the current WHO classification separates TFH-derived PTCL, previously classified as PTCL, NOS, based on the expression of at least two T_FH_-associated antigens (i.e. CD10, CD279/PD-1, Bcl-6, CXCL13, ICOS, SAP, or CXCR5) (4, 28).

**Figure 1:**
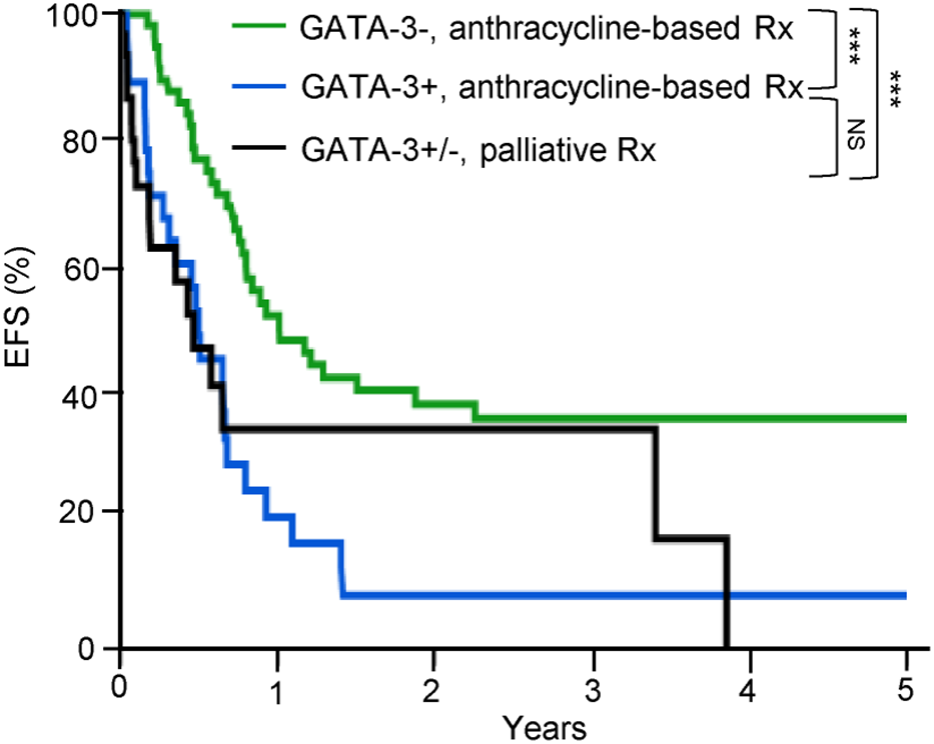
GATA-3 expression is associated with dismal outcomes in PTCL, NOS. GATA-3 expression was determined by immunohistochemistry and patients were stratified by treatment. Among GATA-3 positive patients (n=28) that received first-line anthracycline-based chemotherapy, 86% received CHOP or CHOEP. Among GATA-3 negative patients (n=61), 87% were treated with CHOP/CHOEP. Consistent with prior studies, GATA-3 expression was associated with inferior event-free survival (EFS). Importantly, no significant improvement in EFS was observed for GATA-3+ patients treated with CHOP/CHOEP in comparison to PTCL, NOS patients (n=33) who received palliative or best supportive care alone (72% hospice care with or without corticosteroids), highlighting the futility of current therapies in this subset. [Data reprinted with permission from (39)].

Consistent with an ontologically informed classification schema, two contemporaneous studies demonstrated that PTCL, NOS may be further sub-classified into those that highly express one of two transcription factors that regulate normal T-cell differentiation. One subset expresses the zinc-finger transcription GATA-3, classically associated with T-helper type 2 (Th2) differentiation, and are enriched for a number of its canonical transcripts (i.e. target genes). A second, more heterogeneous subtype(s) is characterized by the expression of the transcription factor T-bet (gene name: *TBX21*), which is classically associated with T-helper type 1 (Th1) and cytotoxic T-cell (CTL) differentiation and function. Iqbal et al. adopted an unbiased approach, profiling 121 PTCL, NOS cases, and observed two dominant subtypes upon unsupervised hierarchical clustering, one enriched for GATA-3, and the other enriched for T-bet transcripts (35). Expression of GATA-3 and T-bet transcripts and protein were inversely correlated (35). A gene expression classifier was able to confidently assign 33% of cases to the GATA-3 group and 49% to the T-bet group, and 18% of cases remained unclassifiable. More recently, PTCL,NOS cases included in this study were utilized as a training cohort to identify an abbreviated molecular classifier (36). This abbreviated molecular classifier, which included 153 transcripts (including 21 viral or housekeeping genes), accurately and reproducibly discriminated “unspecified” from “specified” PTCL subtypes, but also discriminated GATA-3 and T-bet PTCL and was generally concordant (concordance 80%) with an immunohistochemistry-based algorithm previously developed by the same group (29). Consistent with prior observations, T-bet PTCL was enriched in cytotoxic T-cell (CTL) related transcripts (35, 37). As the transcripts utilized in the molecular classifiers were not disclosed, interpreting this work within a broader context is challenging. In contrast, Wang et al. adopted a biased approach after observing evidence for cytokine-driven alternative macrophage polarization in PTCL. Transcriptional profiling of cytokines, including those regulated by T-bet (e.g. IFN-γ) and GATA-3 (e.g. IL-4/IL-13), identified two distinct clusters (37). GATA-3 expression, demonstrated by immunohistochemistry, identified a distinct subset in a multicenter cohort of PTCL, NOS patients, and was associated with inferior progression-free and overall survival. In fact, not a single long-term, disease-free survivor was observed in the GATA-3 group (37), as primary refractory disease is frequently observed in these patients (38). Strikingly, a multicenter study failed to observe a significant difference in survival when comparing patients within the GATA-3 group that had been treated with an anthracycline-based regimen (most commonly CHOP or CHOEP) with those that had received supportive care alone (most commonly hospice) [**Figure 1** (39)].

The contrasting genetic landscape observed in “GATA-3 PTCL” and “T-bet PTCL” provides further evidence that these are truly distinct PTCL subtypes, and should be classified as such, although the current WHO and ICC classifications view the current evidence as insufficient for such a subclassification (4, 40, 41). For example, copy number gains or amplifications involving c-Myc, STAT3, EZH2, and Rel, among others, were recurrent in (and specific for) the GATA-3 subgroup, whereas losses involving the tumor suppressors p53, PTEN, and CDKN2A/B were observed (42). T-cell receptor signaling plays an important role in upregulating GATA-3 protein expression in both conventional and malignant T cells in a PI3K/AKT dependent manner (38, 43), suggesting that PTEN loss may promote translation of GATA-3 transcripts in these lymphomas. Signaling pathways influenced by the aberrant loci observed included PI3K/mTOR and T-cell receptor dependent signaling, consistent with prior studies (35, 38). The distinct, and “high-risk”, genetic landscape associated with GATA-3 PTCL has led some to suggest that GATA-3 does not have an independent oncogenic role, but is simply a surrogate biomarker for a genetically high-risk PTCL subset. However, a comprehensive and systematic analysis of GATA-3 target genes in malignant T cells demonstrated that GATA-3 targets (e.g. including c-myc) are oncogenic, and further demonstrated *via* loss-of-function and gain-of-function studies that GATA-3 is a bona fide proto-oncogene in these lymphomas (39). While less aberrant, distinct and recurrent copy number alterations were observed in T-bet PTCL, including gains involving BCL11B (42), which impairs GATA-3 (Th2) dependent cytokine gene expression upon binding GATA-3 (44). Compared to GATA-3 PTCL, recurrent copy number losses were infrequently observed in T-bet PTCL, although relevant focal losses affecting relevant genes, including TNFAIP3, for example, were occasionally observed. Collectively, the transcriptional and genomic differences observed between GATA-3 and T-bet PTCL demonstrate that these are genetically distinct PTCL subtypes. Furthermore, the high-risk genetic landscape observed in GATA-3 PTCL, including 17p/TP53 deletions and mutations, collectively observed in ≈50% of GATA-3 PTCL, may explain, at least in part, the chemotherapy resistance and dismal outcomes observed in this subgroup.

In contrast to GATA-3 PTCL, the T-bet subgroup is more heterogeneous, and includes minor subsets resembling, and likely derived from, γδ T cells and conventional cytotoxic T cells (CTL) (31–33), including a subset with mutations preferentially affecting epigenetic modifiers, including the DNMT3A methyltransferase domain, leading to hypomethylation of the *EOMES* locus and increased eomesodermin expression, culminating in the induction of a cytotoxic T-cell transcriptional program (31). DNMT3A mutated cases were associated with dismal outcomes, but the inferior survival observed was restricted to T-bet PTCL. While DNMT3A mutations were also observed in GATA-3 PTCL, those mutations did not involve the methyltransferase domain, and were of no prognostic significance. TET2 mutations are also recurrent in T-bet PTCL, and rarely (prevalence <10%) observed in GATA-3 PTCL (36, 45). A rare subset of CTL-derived PTCL, NOS harboring a recurrent IRF4 translocation have also been described (46). In addition, EBV-associated, nodal PTCL, are rarely observed, and usually express CD8 and cytotoxicity-related proteins (45, 47). These findings further highlight the genetic disparities between GATA-3 and T-bet PTCL, but also highlight the heterogeneity within the T-bet subgroup, which is likely comprised of, at the very least, both Th1- and CTL-related lymphomas. Efforts to discriminate these PTCL, NOS subtypes using immunohistochemistry-based algorithms, analogous to those utilized in the classification of diffuse large B-cell lymphoma subtypes, are ongoing (29). The Amador algorithm, for example, includes stains for GATA-3, CCR4, T-bet, and CXCR3, and accurately classified 85% of PTCL, NOS cases when compared with a transcriptionally defined classifier (29). Of course, this ongoing work has significant therapeutic implications, as PTCL, NOS subsets likely have different dependencies, and thus varying degrees of vulnerability to both conventional and novel agents. In fact, the dismal outcomes observed in GATA-3 PTCL, as defined by immunohistochemical staining for GATA-3 alone, following multiagent, anthracycline-based chemotherapy may suggest that this approach is futile in these patients (37, 39, 48, 49). While acknowledging the limitations of making cross-cohort comparisons, it is notable that the outcomes observed in these studies is comparable to those observed in a genetically high-risk subset of PTCL, NOS, defined by TP53 and/or CDKN2A deletions/mutations (>50% of which were biallelic/homozygous) (16). While not reported, the constellation of copy number alterations observed and the prevalent GATA-3 expression reported in this TP53/CDKN2A-altered group (or “group 2”, as defined by Watatani et. al.) would suggest that this group, if transcriptionally profiled, would likely be classified as falling within the GATA-3 subtype (16). Given the anticipated prevalence of TP53/CDKN2A alterations in GATA-3 PTCL (≈50%), these findings may further suggest that GATA-3 expression itself is an adverse prognostic factor, irrespective of the underlying genetic landscape (or at least TP53/CDKN2A status). This contention is supported by the recent observation that GATA-3 itself functions as a proto-oncogene (39), and is consistent with its role in the differentiation, homeostatic survival, and proliferation of conventional (non-malignant) T-cell subsets (50–58). These recent findings also have therapeutic implications, as GATA-3 target genes, including ITK, are targetable (39).

## PTCL, NOS pathogenesis

**Figure 2:**
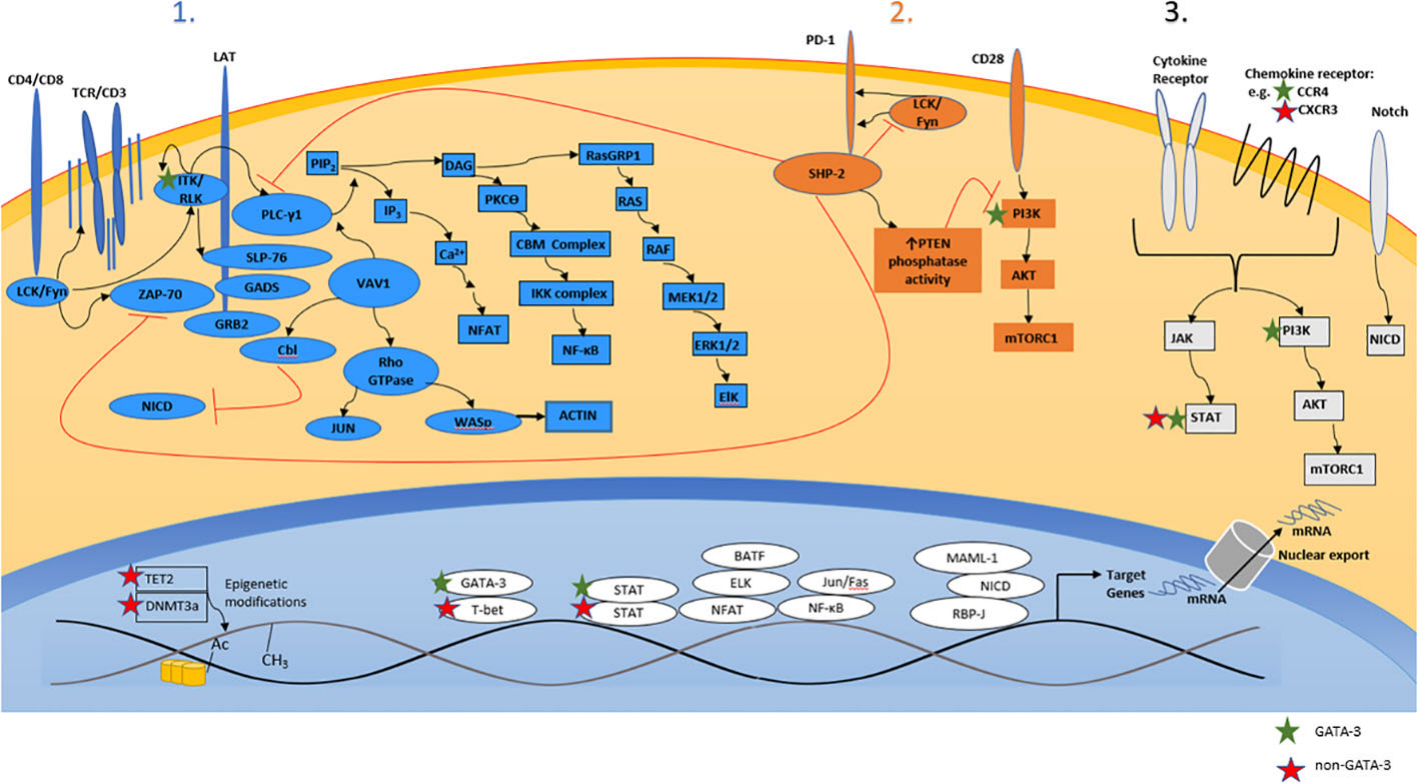
A three-signal model of T-cell lymphomagenesis. Ligands and cytokines/chemokines provided by constituents of the tumor microenvironment engage three broad classes of cell-surface receptors expressed by malignant T cells, including the T-cell receptor (“signal 1”), costimulatory/coinhibitory receptors (“signal 2”), and cytokine/chemokine receptors (“signal 3”). Receptor engagement instigates a signaling barrage culminating in significant transcriptional changes that regulate the proliferation, survival, cytoskeletal remodeling, and metabolism of malignant T cells. Relevant primary/secondary messengers and transcription factors are shown, and those that are preferentially expressed (or activated) in GATA-3 or non-GATA-3 PTCL are indicated.

The antigen-, costimulatory-, and cytokine-dependent signals that regulate the differentiation, proliferation, survival, and function of conventional (non-malignant) T cells are co-opted by malignant T cells during T-cell lymphomagenesis (**Figure 2**). Ligands and cytokines, provided by constituents of the tumor microenvironment (TME), instigate signaling cascades that metabolically and transcriptionally regulate malignant T cells. The antigen-, costimulatory-, and cytokine-receptor dependent signaling inputs provided by the TME regulate and activate transcription factors that regulate target genes with either a cell-autonomous and/or non-cell-autonomous role in T-cell lymphomagenesis. Of course, the repertoire of target genes regulated in this manner is not easily extrapolated from our understanding of conventional (non-malignant) T cells, as transcriptional reprogramming is highly context dependent, being determined by the enhancer and epigenetic landscapes, both of which, in comparison to their conventional counterparts, are altered in malignant T cells (16, 42, 59, 60). Target genes with a cell-autonomous role may cooperate with recurrently altered oncogenes (e.g. c-myc, STAT3) and tumor suppressors (e.g. p53, CDKN2A), promoting the growth and survival of malignant T cells. Conversely, target genes with a non-cell-autonomous role (e.g. cytokines/chemokines) regulate the recruitment, homeostatic survival, expansion, and functional polarization of constituents of the TME. These distinctions can be arbitrary, and are by no means mutually exclusive. For example, CSF-1, a critically important homeostatic cytokine for tissue-resident macrophages, may also activate malignant T cells in an autocrine manner upon binding its receptor (i.e. CSF-1R), which is aberrantly expressed by a subset of PTCL, NOS, culminating in PI3K/AKT activation (61). This model of T-cell lymphomagenesis, described as a “three signal” model [**Figure 2** (62)], has been recently reviewed (63, 64), and is consistent with the genetic landscape associated with PTCL, NOS, as many of the recurrent copy number variants and mutations observed in these lymphomas regulate signaling cascades normally associated with antigen-, costimulation-, or cytokine-receptor dependent signaling. While many gain-of-function mutations may render cells independent from exogenous, ligand-dependent signaling, this is not universally true, and thus a three-signal model also highlights the TME’s supportive role in disease pathogenesis. Conversely, receptors (e.g. Notch) that are rarely subject to mutations or copy number alterations in mature T-cell lymphomas (in contrast to T-ALL) (42, 65), remain dependent upon ligand- and TME-dependent signaling (66). Finally, the signaling cascades triggered by exogenous and TME-dependent factors, and those activated by gain-of-function genetic alterations, confer sensitivity (or resistance) to novel, targeted agents. Consequently, our current, three-signal understanding of PTCL, NOS pathogenesis has significant therapeutic implications.

Analogous to the pathogenic role of “chronic active” and “tonic” B-cell receptor (BCR) signaling in many B-cell lymphomas (67), malignant T cells seemingly exploit T-cell receptor (TCR) dependent signals (38). Just as the genetic landscape observed in B-cell lymphomas supports the BCR’s pathogenic role, activating translocations and mutations involving signaling intermediates downstream of the TCR are recurrently observed in PTCL, NOS. For example, gain-of-function mutations in Src family kinases (SFK), including Fyn, are observed in PTCL, NOS (prevalence <5%). These mutations predominantly disrupt inhibitory interactions between the Fyn SH2 domain and its c-terminal domain by rendering Fyn resistant to Csk-dependent phosphorylation (68). More recently, rare translocations involving the SFK genes for Fyn and Lck have been described (69). Among these, a FYN-TRAF3IP2 fusion, which juxtaposes the Fyn membrane localization (and SH3) domains (but not the kinase domain) with almost the complete open reading frame of TRAF3IP2 (including its TRAF6 binding domain), was most prevalent. TRAF3IP2, which normally activates NF-kB and MAPK pathways downstream from IL-17 receptor signaling, activates these pathways in an IL-17 independent manner. When ectopically expressed in a T-ALL cell line, this fusion led to constitutive NF-kB (but not MAPK) activation. Activation of proximal kinases required for TCR signaling (e.g. Zap-70) was not observed, and NF-kB activation occurred independently from formation of the CARD11/BCL10/MALT1 (CBM) complex, yet TCR activation further increased NF-kB activation in these cells. Therefore, while this novel fusion “hijacks” TCR-dependent NF-kB signaling, further amplification of signaling following TCR activation was observed in these cells. In contrast, a novel (and rare) KHDRBS1-LCK fusion, which includes the Lck kinase domain, increased proximal (and SFK-dependent) TCR signaling, resembling “chronic active” antigen-receptor signaling.

Vav1, mutated in ≈10% of PTCL, NOS (16, 70, 71), is a multidomain and multifunctional protein, with both guanine nucleotide exchange factor (GEF)-dependent and GEF-independent functions, that amplifies and diversifies TCR signaling. In addition to preferentially activating the Rho family GTPase Rac1 and remodeling the actin cytoskeleton, Vav1 also activates NFAT and promotes the ubiquitin-dependent degradation of the Notch intracellular domain independently from its GEF activity. In addition, optimal NFAT activation, even in cells that express constitutively active Vav1, requires TCR activation (72). Systematic interrogation of the broad spectrum of Vav1 mutations (and translocations) observed in many cancer types suggest that approximately 50% of Vav1 mutations are non-functional passenger mutations (73). Among the remaining mutations (and translocations) that are functional, a subset (or so-called “trivalent” mutations) activates both Rac1 and NFAT, but also eliminates Notch inhibitory functions associated with wildtype Vav1. As a class, these trivalent mutations are translocations or truncating mutants that involve the c-terminal SH3 domain, and are commonly observed in PTCL, NOS, but are quite distinct from a class of monovalent mutations that, despite eliminating Notch inhibitor capacity, preserve Rac1 and NFAT activating capacity, and are more commonly observed in alternative T-cell lymphomas (including CTCL, AITL, and ATLL). A less common bivalent class of gain-of-function (GOF) missense mutations observed in multiple Vav1 domains culminate in Rac1 and NFAT activation, but do not affect Notch inhibition, and are also observed in PTCL, NOS (and other T-cell lymphomas). Selected Vav1 mutants and fusions lacking the c-terminal SH3 domain, when transgenically expressed in mouse T cells, lead to the development of PTCL resembling nodal T_FH_-derived lymphomas (73). However, the expression of similarly mutated Vav1 in a p53-deficient context did not lead to the development of PTCL resembling nodal T_FH_-derived lymphomas (74). Instead, the PTCL that emerged in transgenic mice highly expressed GATA-3 and transcriptionally resembled GATA-3 associated PTCL, NOS, and resembling their human counterparts (42), marked c-Myc pathway activation was also observed in these lymphomas. Collectively, these observations highlight the cooperativity between specific mutations (and presumably the pathways they activate) and the broader genetic landscape across the spectrum of PTCL. To date, very few PTCL, NOS cases, stratified into GATA-3 and non-GATA-3 subtypes, have been sequenced. Therefore, the mutational spectrum, at least within the context of a “three signal” model, and its potential relationship to both the putative cell-of-origin and the broader genetic landscape within these ontologically defined PTCL, NOS subsets, remain a significant gap in knowledge.

Consistent with the three-signal model previously proposed, copy number alterations and GOF mutations are recurrently observed in other TCR (signal 1)-related enzymes and adaptors (e.g. PLCγ, CARD11), and either costimulation (signal 2)-related (e.g. ICOS-CD28 fusions, CD28 mutations) or cytokine (signal 3)-related (e.g. JAK2, JAK3, STAT3, SOCS1) genes (16, 42, 75, 76). As noted, the relationship between this mutational spectrum, PTCL, NOS ontology and classification, and the broader genetic landscape, are not yet fully defined. Furthermore, improved understanding of poorly understood or previously uncharacterized drivers may lead to further additions to, or refinements of, our current understanding of T-cell lymphomagenesis and unveil novel therapeutic targets. For example, ligand-dependent Notch signaling was recently discovered as an important driver of proliferation in PTCL, NOS (66).

Constituents of the TME play a direct role in PTCL pathogenesis, by providing ligands/cytokines that engage antigen, costimulatory, and cytokine receptors expressed by malignant T cells, but also play an indirect role, by suppressing host anti-tumor immunity (62). Macrophages, for example, are constituents of the TME in PTCL, NOS (37, 77), and when abundant are associated with poor outcomes (48). In contrast, abundant B-cells and/or dendritic cells within the TME may be associated with more favorable outcomes (77). PTCL-derived and subtype-specific cytokines promote the recruitment, expansion and functional polarization of TME constituents. Macrophages, for example, are characterized by considerable plasticity, and under the influence of GATA-3 dependent cytokines highly express PD-L1 (17, 37), and other immune checkpoints (77), and foster immune evasion and disease progression (78). Given the importance of the TME in PTCL pathogenesis, novel therapeutic strategies to functionally attenuate, exploit, or even deplete constituents of the TME are being actively investigated. The extent to which the microenvironmental ecosystem diverges across, or is defined by, PTCL, NOS subtypes, and its relative contribution to disease pathogenesis, or utility as a therapeutic target, are poorly understood, but important questions.

## Frontline Treatment

As previously noted, the use of anthracycline-based regimens (usually CHOP) in the frontline setting is not only an extrapolation from the treatment paradigm utilized in the management of aggressive B-cell lymphomas, but is not curative for most PTCL, NOS patients. In fact, a recently reported retrospective study demonstrated that survival was dismal for patients with GATA-3 PTCL (as defined by immunohistochemistry) whether they received CHOP/CHOEP or supportive care alone (39). And, more to the point, there was no significant difference between the two groups. Attempts to improve a suboptimal backbone by adding additional agents (79), even a relatively effective one [e.g. brentuximab vedotin (2)], have failed to improve outcomes in PTCL, NOS. For example, the ECHELON-2 trial randomized patients to receive either brentuximab vedotin (BV)-CHP or CHOP. Notably, ≈75% of those enrolled were ALCL patients (2), for whom the overall response rate with single-agent brentuximab vedotin exceeds 80% (80). While a significant survival benefit was observed in the overall study population treated with BV-CHP, this benefit was likely restricted to patients with ALCL. While underpowered to address the potential benefit of BV-CHP in PTCL, NOS, no significant difference in progression-free [hazard ratio: 0.79, 95% CI 0.43-1.43] or overall survival [hazard ratio 0.75, 95% CI 0.37-1.48] was observed among the 72 PTCL, NOS patients randomized (81). Consistent with prior studies (82), CD30 expression among these patients was highly variable (ranging from 10-100%, with a median of 25%) and no correlation with response was observed when using a median cut-point for CD30 expression (81), consistent with prior observations (83).

Consolidation with high-dose therapy followed by autologous stem cell transplantation (HDT-ASCT) in first remission following frontline therapy is associated with ≈40-50% event-free survival at 24 months in single-arm and registry studies (10, 84–86). In contrast, a retrospective study (LYSA) failed to demonstrate a significant benefit associated with HDT-ASCT in an intention-to-treat analysis, but it is notable that 16% within the transplantation arm never proceeded to transplant, often due to primary refractory disease (87). Consequently, anthracycline-based regimens (commonly CHOP or CHOEP), despite uncertainty and a paucity of high-level evidence, and consolidation with high-dose therapy and autologous stem cell transplantation in first remission, remain the cornerstone of treatment for many patients, and is an approach consistent with current treatment guidelines. While this approach may be a “standard of care”, it is not curative for most patients, and primary refractory disease remains a significant challenge (38, 39). Therefore, studies investigating combinatorial and rationally designed strategies using novel and immunomodulatory agents with significant single-agent and/or synergistic activity are most certainly needed (1). The transcriptional and genetic heterogeneity increasingly appreciated across the PTCL, NOS spectrum suggests that the identification of relevant biomarkers in future studies ought to facilitate a more personalized approach, and one that will hopefully improve our ability to pair the “right” – and rationally designed – treatment with the “right” patient.

## Treatment of relapsed and refractory PTCL, NOS

Outcomes for patients with relapsed/refractory PTCL, NOS are dismal, as a median event-free and overall survival <6 months is reasonably anticipated (88, 89), and salvage therapies, short of HDT-ASCT or allogeneic stem-cell transplantation, are largely palliative. Analogous to the approach adopted in the frontline setting, traditional salvage regimens used in the setting of relapsed aggressive B-cell lymphomas (e.g. ICE, DHAP) were often utilized in the setting of relapsed/refractory PTCL, NOS prior to the advent of the novel agents (belinostat, brentuximab vedotin, pralatrexate) currently approved in the United States. The British Columbia Cancer Agency reported their experience in relapsed/refractory PTCL, 52% of which were PTCL, NOS, who did not undergo transplant at relapse (82). Among these patients, 58% (n=89) received either combination regimens or single-agent chemotherapy, including GDP (n=19), non-gemcitabine containing multiagent regimens (e.g. ICE, n=22), and various single agents (e.g. etoposide, gemcitabine, n=48). The median overall survival in the entire cohort was 3.7 months, and was only marginally improved for those receiving chemotherapy, at 6.5 months. As this retrospective study included patients treated prior to the widespread availability of novel agents, including pralatrexate and romidepsin, the median progression-free survival (PFS) following salvage chemotherapy was compared to that observed in phase II studies with pralatrexate or romidepsin (90, 91), and median PFS of 3.7, 3.5, and 4 months were observed, respectively (88). Furthermore, exceptional, durable responses may be achieved with novel agents, particularly HDAC inhibitors. Among 130 PTCL patients treated with romidepsin, 10 patients (5 with PTCL, NOS) achieved a durable (>12 months) and complete response, 6 of whom (3 with PTCL, NOS) remained on treatment >2 years (92). Given the favorable toxicity profile associated with novel agents, many patients are exposed to these agents in a sequential manner. The exceptional responses observed in selected patients, and the ability to provide treatment in a sequential manner for many others, may explain the improved overall survival observed with single, novel agents when compared with more toxic, multiagent regimens in retrospective studies (9, 89, 93). For transplant eligible patients who did not undergo consolidation with HDT-ASCT in first remission, HDT-ASCT among those responding to salvage therapies is potentially curative, with 3-year OS ≈50% (94, 95). Allogeneic stem cell transplantation, while associated with significant transplant related-mortality, is also a potentially curative approach for eligible patients (94, 95). Autologous and “off-the-shelf” allogeneic cellular therapies, including CAR-T products specific for T-cell specific antigens, are an active area of ongoing investigation [reviewed in (96)].

Given the advances achieved over the past decade, the PTCL are anything but an “unexplored frontier”. In fact, the dramatic expansion in the development of targeted agents, and the sheer number of possible doublet (and triplet) combinations incorporating them, has seemingly outstripped the capacity for their methodical interrogation in a rare disease. This significant challenge is only exacerbated by recognition of the divergent genetic landscape and disease ontogeny between GATA-3 and the more heterogeneous non-GATA-3 subsets, and should be accounted for in future clinical trials using novel agents. While a systematic review of those agents currently under investigation is beyond the scope of the present review, a summary is provided in **Table 1**. However, it is notable that approximately one-third of the trials reviewed failed to discriminate between PTCL, NOS and other PTCL subtypes. Similarly, among the almost 500 PTCL, NOS patients reported in the remaining clinical trials, the distinction between GATA-3 and non-GATA-3 (T-bet) PTCL is unknown. Given the stark – and therapeutically significant – differences between these PTCL subtypes, this obvious gap in knowledge must be addressed in future studies. In contrast to the road previously travelled, the path forward will increasingly rely on an improved understanding of PTCL, NOS pathogenesis, and clinical trial participation – perhaps like never before – will remain a critically important consideration in the management of these patients, as we believe targeted agents and novel combinations targeting both malignant T cells and constituents of their TME are particularly promising.

**Table 1:** Novel agents utilized in PTCL, NOS.

Drug	MOA	ORR (PTCL, NOS)	ORR (non-PTCL, NOS)
**Bendamustine** (97)	Alkylating agent	30/60 (50%)**	AITL 22/32 (69%)
**Gemcitabine** (98)	Nucleoside analog	11/20 (55%)	MF 9/19 (48%)
**5-azacytidine** (99)	Nucleoside analog	N/A (not eligible)	AITL 9/12 (75%)
**Guadecitabine** (100)	Hypomethylating agent	0/2 (0%) 8/20 (40%)***	TFH 7/16 (44%)
**Pralatrexate** (90)	Dihydrofolate reductase inhibitor	19/59 (32%)	AITL 1/13 (8%) ALCL 6/17(35%)
**Fenretinide** (101)	Synthetic retinoid	0/1 (0%)	AITL 2/3 (66%) CTCL 2/6 (33%) Gamma-Delta TCLs 0/1 (0%)
**Belinostat** (102)	Histone deacetylase (HDAC) inhibitor	18/77 (23%)	AITL 10/22 (46%) ALK- ALCL 2/13 (15%) ALK+ ALCL 0/2 (0%)
**Romidepsin** (91)	Histone deacetylase (HDAC) inhibitor	20/69 (29%)	AITL 8/27 (30%) ALK- ALCL 5/21 (24%)
**Tucidinostat** (103)	Histone deacetylase (HDAC) inhibitor	12/34 (35%)	AITL 7/8 (87%) ALK- ALCL 1/3 (33%) EATL 1/1 (100%)
**Chidamide** (104)	Histone deacetylase (HDAC) inhibitor	6/27 (22%)	ENKL 3/16 (18%) ALK- ALCL 5/11 (45%) ALK+ ALCL 2/6 (33%) AITL 5/10 (50%)
**Duvelisib** (105)	PI3K inhibitor	8/16 (50%)***	CTCL 6/19 (31.6%)
**Linperlisib** (106)	PI3K inhibitor	6/12 (50%)	AITL 8/10 (80%)
**Tenalisib** (107)	PI3K inhibitor	7/15 (46%) ***	CTCL 9/20 (45%)
**Copanlisib** (108)	PI3K inhibitor	5/10 (50%)***	
**Enzastaurin** (109)	Serine/threonine kinase inhibitor	0/13 (0%)***	CTCL 1/11 (9%)
**Alisertib** (110)	Aurora A kinase inhibitor	34/102 (33%)**	
**Everolimus** (111)	mTORC1 inhibitor	3/4 (75%)	CTCL 3/7 (43%)
**Ruxolitinib** (112)	Janus Kinase (JAK) inhibitor	2/11 (18%)	TPLL 3/8 (37%) TFH 3/9 (33%) T-LGL 2/5 (40%) ALCL 1/4 (25%) MF 1/7 (14%) Gamma-Delta TCLs 1/4 (25%)
**Golidocitinib** (113)	Janus Kinase (JAK) inhibitor	5/19 (26%)	AITL 13/20 (65%) ALK- ALCL 2/4 (50%) NKTCL 1/4 (25%)
**Cerdulatinib** (114)	SYK/JAK inhibitor	9/38 (35%) *	AITL 12/22 (55%)
**Cpi-818** (115)	Interleukin-2-Inducible T-cell Kinase (ITK) inhibitor	1/4 (25%)	CTCL 1/7 (14%)
**Bortezomib** (116)	Proteasome inhibitor	1/2 (50%)	MF 7/10 (70%)
**Ixazomib** (117)	Proteasome inhibitor	1/2 (50%)	CTCL 0/5 (0%) ALK- ALCL 0/2 (0%) TFH 0/3 (0%)
**Tolinapant** (118)	Antagonist of the cellular and X-linked inhibitor of apoptosis proteins (cIAP1/2 and XIAP)	21/98 (21%) ***	CTCL 13/50 (26%)
**Forodesine** (119)	Purine nucleoside phosphorylase inhibitor	5/22 (23%)	AITL 6/18 (33%)
**Ibrutinib** (120)	Bruton Tyrosine Kinase (BTK) inhibitor	0/3 (0%)	MF 1/6 (16%)
**Valemetostat** (121)	EZH1/EZH2 inhibitor	24/44 (55%) ***	ATLL 8/14 (57%)
**Imatinib mesylate** (122)	Platelet derived growth factor inhibitor	0/12 (0%)	
**Tipifarnib** (123)	Farnesyltransferase inhibitor	4/10 (40%) Note: PTCL CXCL12 chemokine receptor +	AITL 18/32 (56%)
**Alemtuzumab** (124)	CD52 monoclonal antibody	3/6 (50%)	CTCL 3/4 (75%)
**Brentuximab vedotin for CD30+** (83)	CD30 monoclonal antibody-drug conjugate	7/21 (33%) Note: CD30+ PTCL NOS	CD30+ AITL 7/13 (54%)
**Camidanlumab tesirine** (125)	CD25 monoclonal antibody-drug conjugate	2/6 (33.3%)	ATLL 3/7 (43%)
**Mogamulizumab** (126)	CCR4 monoclonal antibody	3/16 (19%)	AITL 6/12 (50%), ALK- ALCL 1/1 (100%) CTCL 3/8 (38%)
**Pembrolizumab** (127)	PD-1 monoclonal antibody	1/5 (20%)	ALCL 1/1(100%) MF 1/3 (33%) FTCL 2/4 (50%) HSTCL 0/1(0%) MEITL 0/1 (0%)
**Nivolumab** (128)	PD-1 monoclonal antibody	2/5 (40%) ***	MF 2/13 (15%)
**TTI-621 (SIRPα-IgG1 Fc)** (129)	Binds CD47	2/11 (18%)***	CTCL 6/29 (21%)
**Lenalidomide** (130)	Immunomodulation	4/20 (20%)	AITL 8/26 (31%)
**Doublets:**			
**Romidepsin + Pralatrexate** (131)		10/14 (71%) **	
**Romidepsin + 5-Azacytidine** (132)		14/23 (61%) ***	TFH 12/15 (80%)
**Romidepsin + Duvelisib** (133)		12/22 (55%) ***	CTCL 6/13 (46%)
**Romidepsin +Tenalisib** (134)		4/5 (80%)	CTCL 8/15 (53%) AITL 4/5 (80%)
**Romidepsin + Lenalidomide** (135)		5/10 (50%)	CTCL 5/9 (56%)
**Duvelisib + Bortezomib** (133)		5/14 (36%) ***	CTCL 4/14 (29%)

*Response among all non-AITL subtypes.

**Response among all T-cell lymphomas (PTCL/CTCL).

***Response among all PTCL subtypes.

AITL, Angioimmunoblastic T-cell lymphoma; ALCL, Anaplastic large cell lymphoma; ALK, Anaplastic Lymphoma Kinase; ATLL, Adult T-cell leukemia/lymphoma; CTCL, Cutaneous T-cell lymphoma; EATL, Enteropathy-associated T-cell Lymphoma; ENKL, Extranodal Natural Killer/T-cell lymphoma, nasal type; FTCL, Follicular T-cell lymphoma; Gamma-Delta TCL, Gamma-Delta T-cell lymphoma; HSTCL, Hepatosplenic T-cell lymphoma; MOA, Mechanism of Action; MEITL, Monomorphic epitheliotropic intestinal T-cell lymphoma; MF, Mycosis Fungoides; NKTCL, Natural Killer/T-cell Lymphoma; ORR, Overall response rates; PTCL, NOS, Peripheral T-cell lymphoma not otherwise specified; TFH, Nodal peripheral T-Cell lymphoma, T-follicular helper phenotype; TLGL, T-cell large granular lymphocytic leukemia; T-PLL, T-cell prolymphocytic leukemia.

## Author contributions

JW, JR, and RW reviewed the literature, organized and wrote the manuscript. All authors contributed to the article and approved the submitted version.
